# Low‐Cost and Detunable Wireless Resonator Glasses for Enhanced Eye MRI With Concurrent High‐Quality Whole‐Brain MRI

**DOI:** 10.1002/mrm.70326

**Published:** 2026-02-24

**Authors:** Ming Lu, Xiaoyue Yang, Jason E. Moore, Pingping Li, Adam W. Anderson, John C. Gore, Seth A. Smith, Xinqiang Yan

**Affiliations:** ^1^ Vanderbilt University Institute of Imaging Science Vanderbilt University Medical Center Nashville Tennessee USA; ^2^ Department of Electrical and Computer Engineering Vanderbilt University Nashville Tennessee USA; ^3^ Philips Nashville Tennessee USA; ^4^ Department of Biomedical Engineering Vanderbilt University Nashville Tennessee USA; ^5^ Department of Radiology and Radiological Sciences Vanderbilt University Medical Center Nashville Tennessee USA

**Keywords:** eye, passive resonator, signal‐to‐noise ratio (SNR), ultrahigh field, wireless resonator

## Abstract

**Purpose:**

To develop a wearable wireless resonator glasses design that enhances eye MRI signal‐to‐noise ratio (SNR) without compromising whole‐brain image quality at 7 T.

**Methods:**

The device integrates two detunable LC loop resonators into a lightweight, 3D‐printed frame positioned near the eyes. The resonators passively couple to a standard 2Tx/32Rx head coil without hardware modifications. Bench tests assessed tuning, isolation, and detuning performance. *B*_1_^+^ maps were measured in a head/shoulder phantom, and SNR maps were obtained in both phantom and in vivo experiments.

**Results:**

Bench measurements confirmed accurate tuning, strong inter‐element isolation, and effective passive detuning. Phantom *B*_1_^+^ mapping showed negligible differences between configurations with and without the resonators. Phantom and in vivo imaging demonstrated up to a ∼3‐fold SNR gain in the eye region, with no measurable SNR loss in the brain.

**Conclusion:**

The wireless resonator glasses provide a low‐cost, easy‐to‐use solution that improves ocular SNR while preserving whole‐brain image quality, enabling both dedicated eye MRI and simultaneous eye–brain imaging at ultrahigh field.

## 1 Introduction

Eye MRI plays a vital role in the non‐invasive assessment of a wide range of ocular and neuro‐ophthalmologic conditions, including optic neuropathies, ocular tumors, retinal abnormalities, and disorders of the visual pathway [1, 2, 3, 4, 5, 6, 7, 8]. MRI offers exceptional soft tissue contrast, high spatial resolution, and the ability to capture both structural and functional information without ionizing radiation, making it particularly advantageous for longitudinal monitoring and pediatric imaging.

Despite these advantages, conventional MRI hardware imposes significant limitations on image quality in the ocular region when standard head coils are used [9, 10, 11, 12]. Head coils are designed with a large inner diameter (especially in the anterior/posterior direction) to accommodate a broad range of adult head sizes and ensure patient comfort. As a result, in most situations, the eyes are positioned several centimeters away from the nearest receive elements, leading to a substantial reduction in signal‐to‐noise ratio (SNR) in the orbital region. Furthermore, the loop elements in head coils are typically much larger than the optimal size for ocular imaging, further limiting SNR performance.

Although dedicated eye coils can significantly improve image quality by placing appropriately sized receive elements in close proximity to the eyes, they are not part of the standard configuration on research or clinical MRI systems [13, 14, 15]. Even when available, dedicated eye coils are typically limited in field of view and solely for the orbit [13, 15], thereby restricting their utility in studies involving the visual pathways or brain.

Meanwhile, numerous structural MRI studies have demonstrated that ocular diseases such as glaucoma, amblyopia, macular degeneration, and hereditary retinal dystrophies are often associated with changes in the optic nerve, visual pathways, and even cortical regions [7, 16, 17, 18, 19, 20, 21, 22, 23, 24, 25]. These findings underscore the need for imaging solutions that not only enhance SNR in the eye but also preserve the ability to perform high‐quality whole‐brain MRI within the same setup.

Previous studies have demonstrated that wireless resonators, as well as metamaterials employing similar mechanisms, can enhance localized sensitivity in the head, temporomandibular joint, carotid arteries, breast, wrist, and knee [26, 27, 28, 29, 30, 31, 32, 33, 34, 35, 36, 37, 38, 39, 40, 41, 42, 43, 44]. However, it remains unclear how much benefit wireless resonator designs can provide for eye MRI and to what extent brain SNR can be maintained, especially when wireless resonators operate synergistically with a local receive head array.

In this study, we propose a novel glasses‐frame design incorporating detunable wireless resonators for eye MRI at 7 T. In this study, we propose a novel glasses‐frame design incorporating detunable wireless resonators for eye MRI at 7 T. The design consists of two wireless resonators; each tuned to the 7 T Larmor frequency of 298 MHz and effectively decoupled using capacitive decoupling. Each resonator includes a passive detuning circuit and is integrated into a 3D‐printed eyewear frame. The resonators are optimally sized for ocular imaging and can be readily positioned close to the eyes to potentially enhance localized sensitivity in the ocular region. At the same time, by retaining the head array as the primary receive coil, this design is intended to preserve brain MRI performance.

## 2 Methods

### 2.1 Hardware Design and Fabrication

**FIGURE 1:**
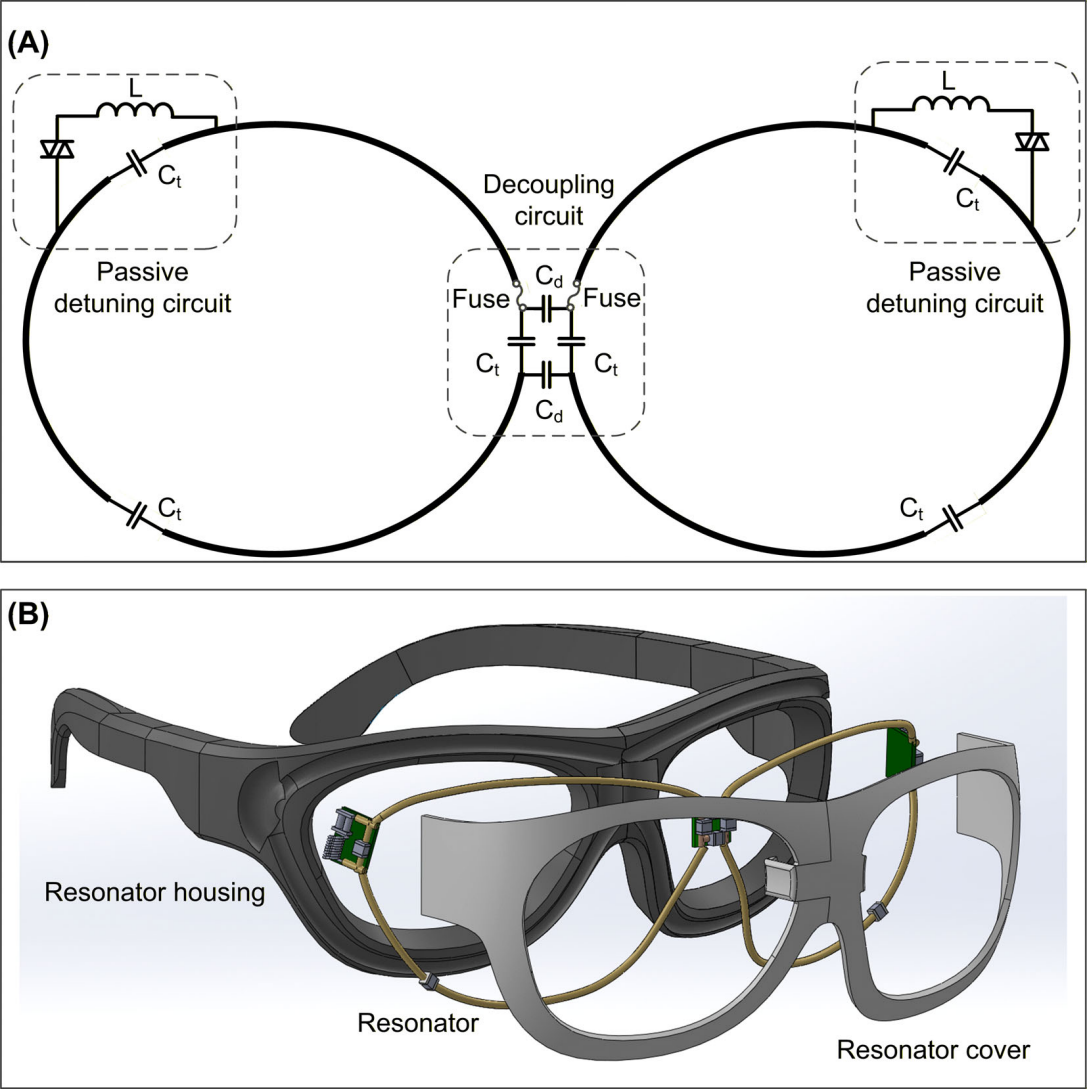
Circuit diagram (A) and CAD design (B) of the wireless resonator glasses.

Figure 1A shows the circuit schematic of the wireless resonator array (hereafter referred to as wireless resonator glasses) designed for eye MRI at 7 T. It consists of two LC loop resonators, each precisely tuned to the Larmor frequency (298 MHz) of our 7 T whole‐body scanner (Philips, Best, Netherland). Each resonator contains three distributed capacitors (labeled *C*_t_ in Figure 1A), arranged to provide at least one capacitor per 1/20 of the wavelength, and is equipped with a passive detuning circuit to ensure automatic deactivation during the transmit (Tx) period. A bridge capacitive network (labeled *C*_d_ in Figure 1A) is used to eliminate mutual coupling between the two loops [45]. For added safety, MRI‐compatible fuses are integrated into each loop to prevent unintended resonant behavior in the event of detuning failure.

This wireless resonator glasses design operates on the same principle as in previous studies [26, 33, 41]: it works in conjunction with the local array during the receive (Rx) period and remains effectively invisible using a passive detune circuit that consists of lumped components during the Tx period. At 7 T, the local array is the Nova volume Tx/32‐channel Rx head coil (Nova Medical, Wilmington, MA, USA), a widely used standard configuration. During the Rx period, the resonators couple passively to the 32‐channel receive array, enhancing local signal reception through close‐proximity inductive coupling without requiring any direct electrical connection or active circuitry.

The two resonators, including tin‐coated wires (AWG 14, diameter 1.63 mm) and components, were embedded within a customized glasses frame, as shown in Figure 1B. The frame was designed in SolidWorks (Dassault Systèmes SE, Vélizy‐Villacoublay, France) and 3D‐printed using a Formlabs 3 printer (Formlabs Inc., Somerville, MA, USA). The frame had an overall width of 15 cm, a height of 6 cm, and a center‐to‐center distance of 7.5 cm between the two resonator positions. The eye region was deliberately left open to ensure compatibility with eye‐tracking systems during MRI.

### 2.2 Bench Test

Bench testing was conducted to ensure that both wireless resonators were properly tuned to the Larmor frequency, were well decoupled, and also were effectively detuned. This test was performed using a pair of well‐decoupled double pick‐up probes. The decoupling performance was assessed by comparing the unloaded quality (*Q*_un_) factor of the two‐resonator system to that of a single ideal resonator, ensuring the merged resonance peak resembled that of a single resonator. Passive detuning performance was also evaluated on the bench by actively turning the cross diodes ON and OFF. While this active switching does not fully represent in‐scan conditions where the diodes are switched passively, it provides a resonant‐based validation of the detuning effectiveness when the diodes are turned ON during the Tx period.

### 2.3 MRI Experiments

MRI experiments were conducted at 7 T with the Nova volume Tx/32‐channel Rx head coil alone, and in combination with the wireless resonator glasses.

To evaluate whether the presence of the wireless resonators influences the transmit (*B*_1_^+^) field generated by the volume transmit coil, *B*_1_^+^ experiments were conducted on a head/shoulder‐shaped phantom. Because the device is intended to operate in receive‐only mode, leaving the resonators partially or fully tuned would allow significant induced currents, which could distort the *B*_1_^+^ field of the volume coil and elevate local SAR [30]. Full detuning ensures RF transparency during the Tx period and avoids transmit‐related safety concerns.

The phantom was fabricated with distilled water, sugar, NaCl, and gel, and was designed to mimic human tissues at 298 MHz, with a measured conductivity of approximately 0.5 S/m and a relative permittivity of ∼55. The *B*_1_^+^ maps were measured using the TurboFLASH method [46], with the same input power. The imaging parameters included a field of view (FOV) of 250 × 250 mm^2^, a slice thickness of 3 mm, and an in‐plane resolution of 3 × 3 mm^2^.

We then assessed the SNR on the head/shoulder gel phantom. SNR maps were calculated from gradient recalled echo (GRE) images with the following parameters: axial orientation, FOV = 250 × 250 mm^2^, TR/TE = 1000/1.96 ms, nominal flip angle (FA) = 70°, in‐plane resolution = 1 × 1 mm^2^, slice thickness = 5 mm, and number of averages = 1. Besides GRE images with a FA of 70°, noise‐only maps were acquired using the exact same parameters, except with the RF power turned off. The noise correlation matrix of the 32‐channel Rx array was calculated based on the noise‐only data, and SNR maps with the optimal combination method were generated based on the GRE images and noise data [47].

In addition to phantom images, T1‐weighted (T1W) and T2‐weighted (T2W) images of a healthy volunteer were acquired using the Nova 2Tx/32Rx coil, without and with the wireless resonator glasses. SNR maps of the human images were calculated based on the scanner's default reconstructed images. The imaging protocols were as follows:

1. T1W imaging: Axial acquisition, TR/TE = 5.0/2.2 ms, FA = 7°, FOV = 220 × 220 × 40 mm^3^, voxel size = 1.0 × 1.0 × 1.0 mm^3^, bandwidth (BW) = 505 Hz/pixel, number of averages = 1;
2. T2W: axial slices, TSE, TR/TE = 3000/302 ms, FA/refocusing FA = 100/35°, FOV = 2770 × 100 × 34 mm^3^, voxel size = 0.7 × 0.7 × 0.7 mm^3^, BW = 1157 Hz/pixel, number of averages = 1.

Safety tests for gradient‐induced and RF‐induced heating were conducted before human imaging. All experimental procedures were approved by the local institutional review board (IRB #060730), and participants provided informed written consent.

## 3 Results

### 3.1 Bench Test Results

**FIGURE 2:**
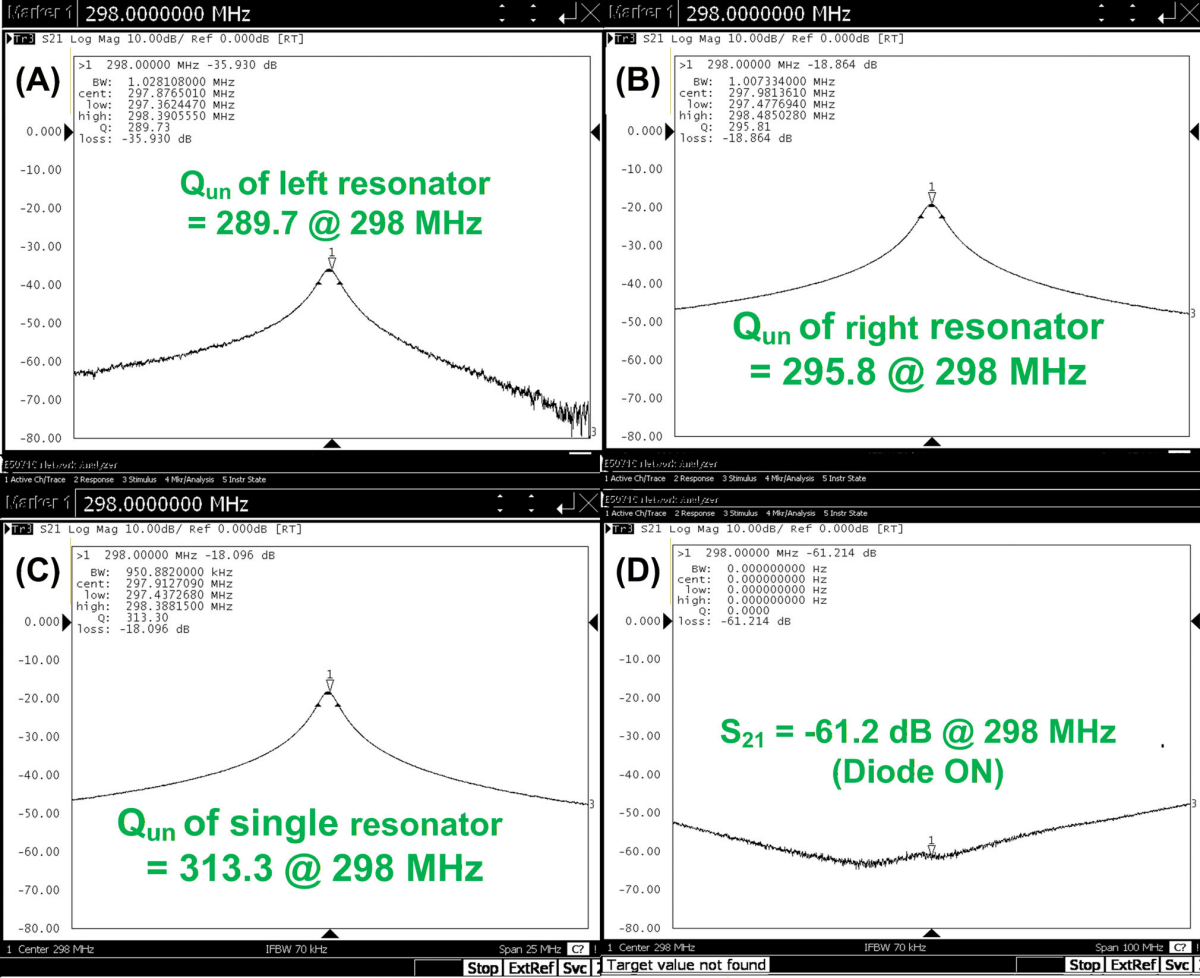
Bench test results under different scenarios using double pickup probes. (A) *Q*_un_ of the left resonator in the wireless resonator glasses. (B) *Q*_un_ of the right resonator. (C) Baseline comparison of the *Q*_un_ of a single resonator, measured without the presence of the other resonator. (D) *S*_21_ measurement of the double probes on the right resonator with the cross diode turned OFF.

Figure 2A,B shows the measured *S*_21_ plots and calculated Q‐factors of the double‐pickup probes when both resonators are well‐tuned to 298 MHz and are well‐decoupled, without any loading. The average *Q*_un_ of these two resonators is 292.8. Additionally, we present the *S*_21_ plot of a single ideal resonator (without the presence of another resonator), which exhibits a *Q*_un_ of 313.3 (Figure 2C). The distinct single‐resonator peak observed in the two‐resonator array, along with the high *Q*_un_ value (93.5% of the *Q*_un_ of a single ideal resonator), demonstrates the excellent isolation and decoupling between the two resonators.

Figure 2D presents the *S*_21_ plots when the cross diodes are actively turned ON. A significant reduction in the *S*_21_ magnitude at 298 MHz (from −30.4 to −61.2 dB) demonstrates the effective detuning capability of the resonator when the cross diodes are activated during RF transmission.

### 3.2 *B*_1_ ^+^ Results

**FIGURE 3:**
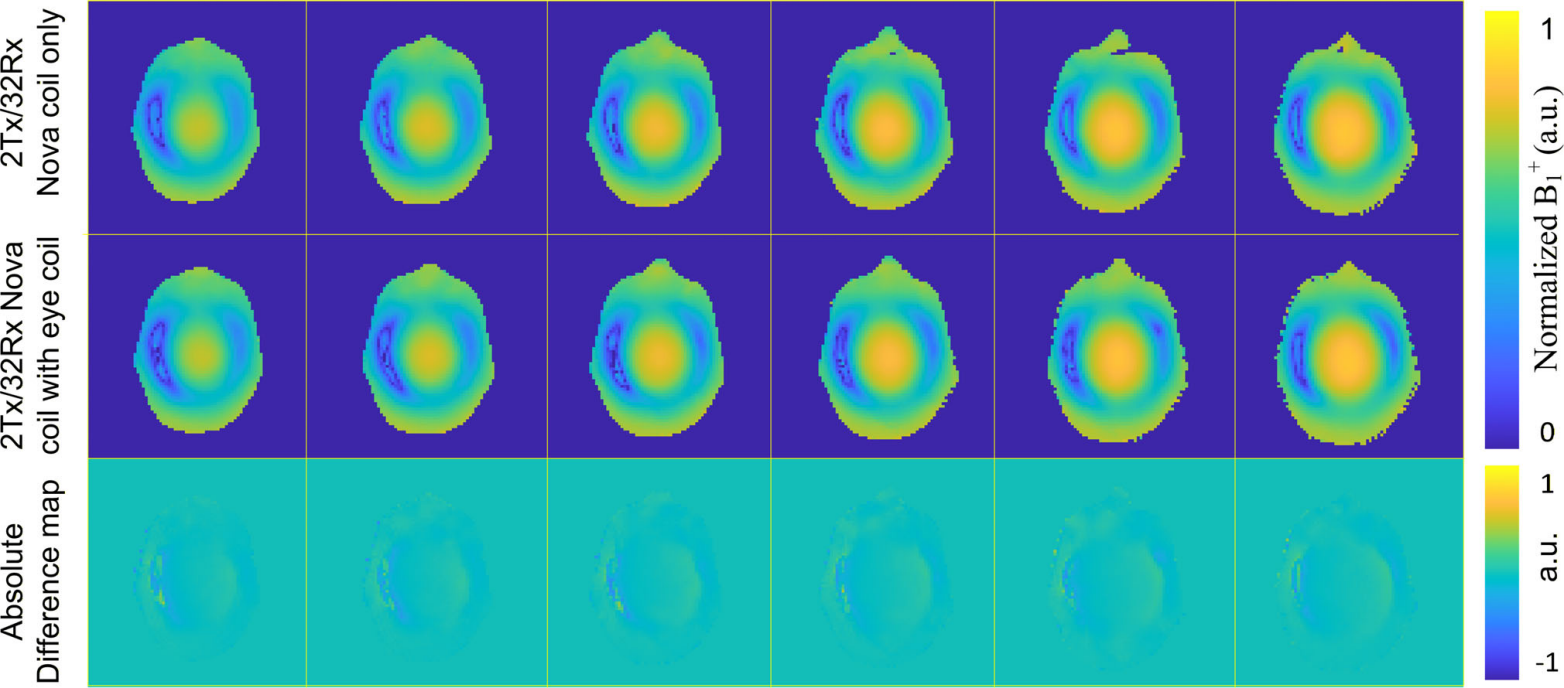
Measured axial *B*_1_^+^ maps of the head/shoulder phantom without (top row) and with (middle row) the wireless resonator glasses, as well as the difference map between the two conditions (bottom row).

Figure 3 shows the measured axial *B*_1_^+^ maps of the head/shoulder phantom acquired using the Nova coil under identical input power for two configurations: without and with the wireless resonator glasses. Both *B*_1_^+^ maps were normalized using the same scaling factor to ensure direct comparability. Under identical input power, the average flip angles across the head‐shaped phantom differed by less than 5% between the two configurations. This outcome aligns with expectations, as the wireless resonators are detunable and remain “invisible” during RF transmission, producing negligible changes in the *B*_1_^+^ field.

### 3.3 Phantom and In Vivo SNR

**FIGURE 4:**
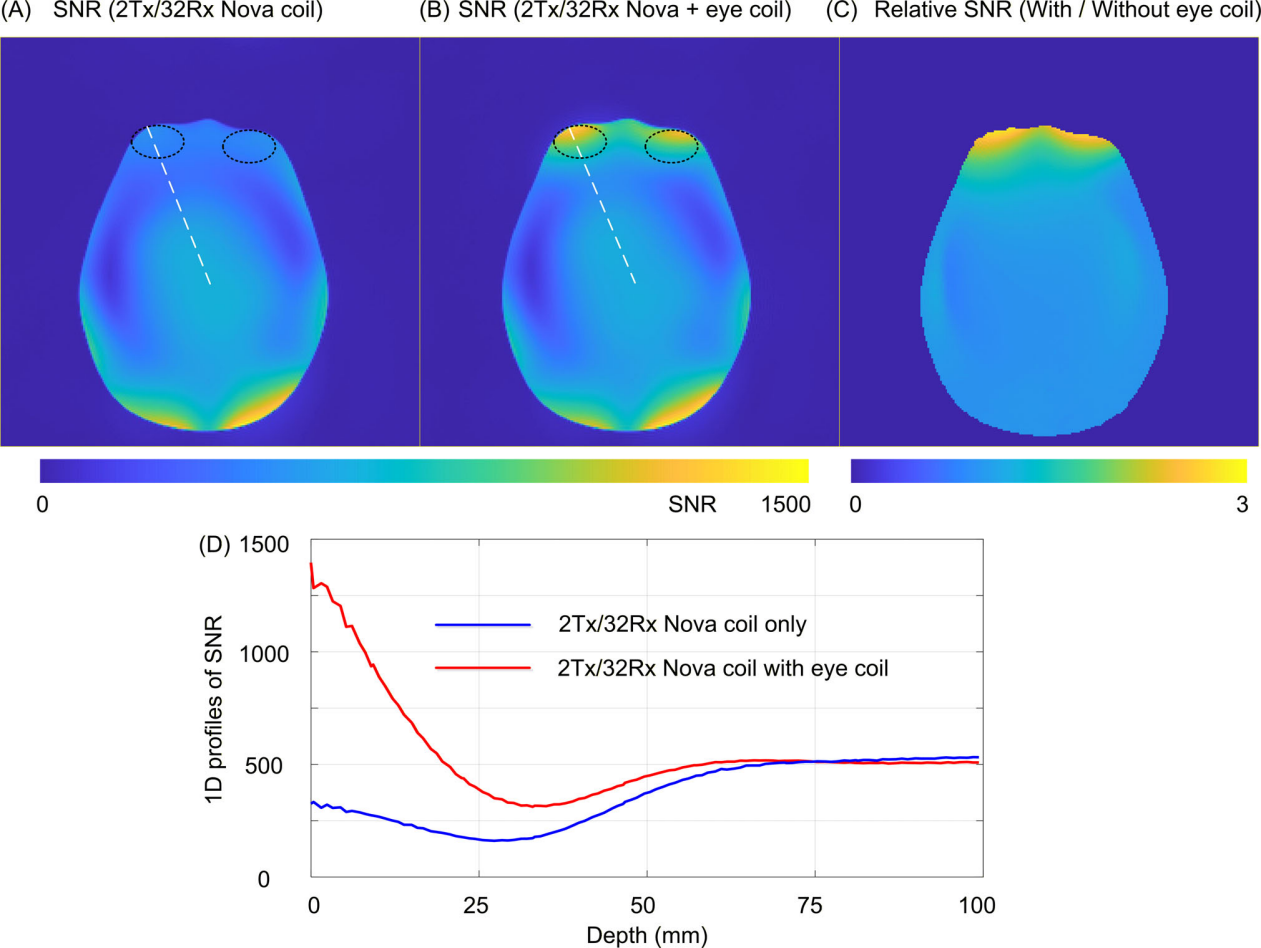
(A) and (B) Axial SNR maps measured without and with the wireless resonator glasses. (C) Corresponding SNR ratios from (A) and (B). (D) 1D SNR profiles along the dotted lines shown in (A) and (B).

Figure 4A,B show axial SNR maps acquired with the Nova 2Tx/32Rx coil without and with the wireless resonator glasses, and Figure 4C shows the corresponding SNR ratios. On average, a 2.6‐fold increase in SNR was observed in the eye region, as indicated by the elliptical circle in the figure. Meanwhile, the SNR in other areas remained at the same level, demonstrating that the wireless resonator can maintain the same level of SNR in the brain area while significantly improving SNR in the eye region. Additionally, a 1D plot of the SNR map was generated (Figure 4D), showing that the SNR benefits are present at depths less than 5 cm but diminish beyond 5 cm in the phantom.

**FIGURE 5:**
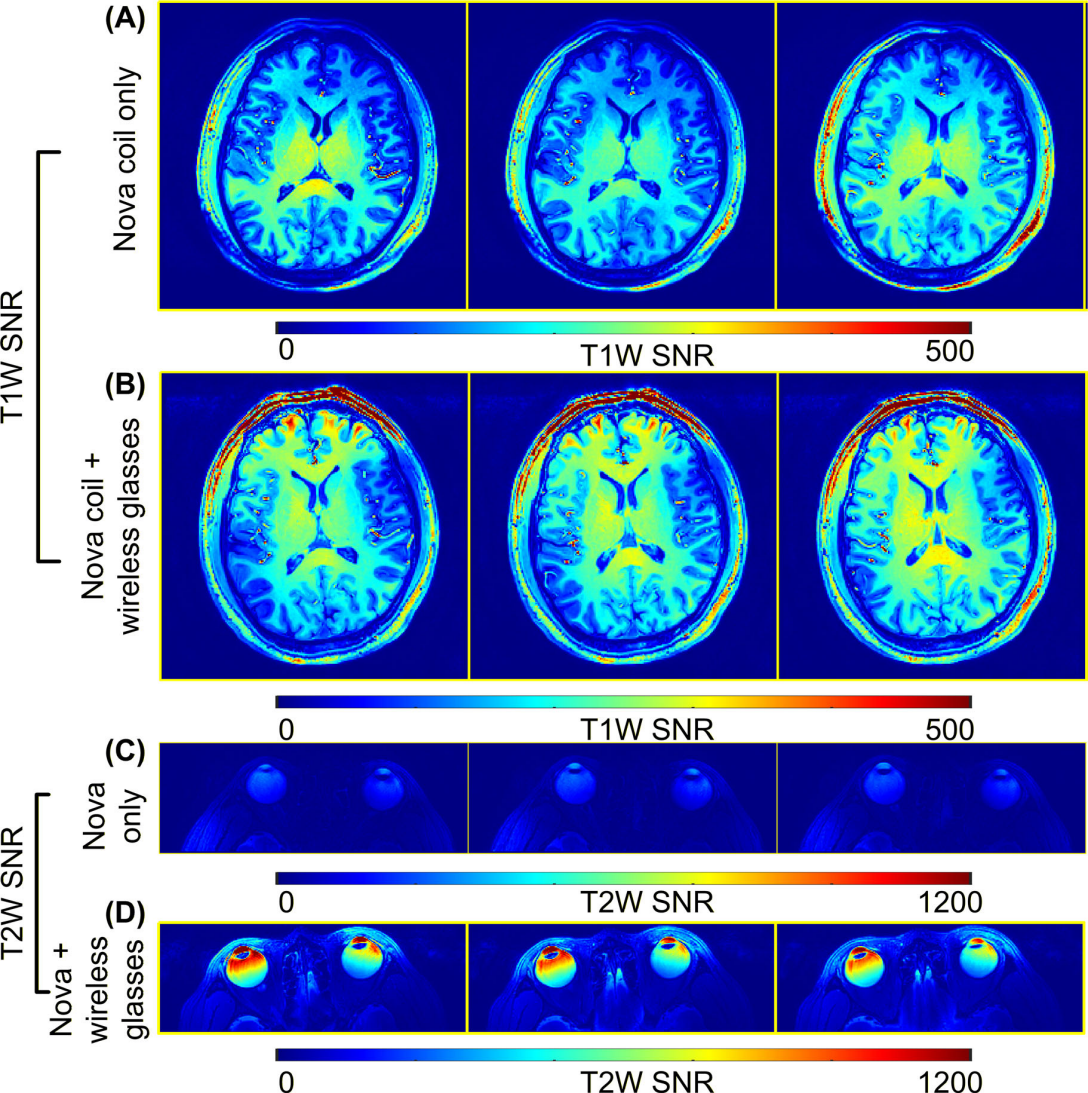
In vivo SNR maps with the Nova 2Tx/32Rx coil based on T1W (A) and T2W (B) images, without and with the wireless resonator glasses. The resonator yielded a ∼3‐fold SNR increase in the eyes and improved SNR in the frontal lobe, while preserving overall brain image quality.

Figure 5A shows the in vivo T1‐weighted whole‐brain images acquired with the Nova 2Tx/32Rx coil, without and with the wireless resonator glasses. Figure 5B presents representative T2‐weighted images focused on the eye region. Consistent with the phantom results, the addition of the resonator produced a localized SNR gain, most pronounced in the eye region, with an average 3.2‐fold increase in SNR.

## 4 Discussions and Conclusion

This study demonstrates the feasibility and effectiveness of a wearable wireless resonator glasses for enhancing SNR in the eye region during MRI without compromising brain image quality. By embedding detunable LC loop resonators into a lightweight, 3D‐printed frame positioned close to the eyes, we achieved up to a ∼3‐fold SNR improvement in the eye region in phantom and in vivo experiments. Importantly, SNR across most of the brain remained unchanged, confirming that the resonator does not compromise overall brain image quality. Furthermore, the frontal lobe also exhibited improved SNR with wireless resonator glasses, likely due to its anatomical proximity to the eyes and resonators. The wireless resonator glasses effectively enhance eye imaging at 7 T MRI by leveraging near‐field sensitivity to achieve high SNR near the resonator surface. The eyeglass‐mounted wireless resonator design principle can be extended to lower field strengths, such as 1.5 T and 3 T, with appropriate adjustments to account for differences in SNR and penetration depth.

From a practical standpoint, the fully 3D‐printed eyeglass features a mechanically compliant frame—optionally equipped with an adjustable elastic band—that accommodates a wide range of head sizes. Integrating the wireless resonators directly into the eyeglass structure ensures that they remain consistently close to the eye surface. Notably, even when the eyeglass frame was displaced by 1 cm farther from the eye, a significant SNR improvement was still observed, as shown in Figure S1. The open‐eye design preserves compatibility with eye‐tracking systems, which is valuable for functional MRI studies involving visual stimuli.

When two resonators operating at the same frequency are placed in close proximity, strong mutual coupling can lead to resonance peak splitting. Therefore, in the presence of a primary receive coil, wireless resonators that share the same LC structure and the same resonance frequency would, in principle, be expected to exhibit resonance splitting. In our previous works [26, 27], we observed that this potential disadvantage was outweighed by the benefits of increased receive sensitivity. Based on our recent findings, we further believe that the low input impedance of modern preamplifiers used in the primary receive coil can effectively suppress resonance peak splitting. Additional details and supporting data are provided in Figure S2.

A direct comparison with a dedicated wired eye coil would be informative. However, because commercial 7 T eye coils are not currently widely available and such a coil was not accessible at our site, a direct experimental comparison was not feasible. While a dedicated eye coil may provide higher SNR than the proposed wireless design in the eye region due to its direct signal reception, the wireless resonator offers several practical advantages, including preserving full‐brain imaging capability while enhancing ocular SNR, as well as lower fabrication cost and simpler deployment. In addition, more extensive quantitative analyses, such as tissue contrast or contrast‐to‐noise ratio measurements across a larger subject population, would be beneficial for better assessing the diagnostic value of this design and represent an important direction for future studies. It should be noted that the proposed wireless resonator glasses are not intended to replace optimized dedicated eye coils, but rather to provide a practical and accessible alternative when such dedicated coils are unavailable.

This work demonstrates promising advancements in wireless resonator design for ocular imaging, but several limitations and areas for improvement should be noted. First, the wearable glasses design adds minimal additional space within the coil. However, the 7 T Nova receive coil itself is relatively compact, and fitting can be challenging for subjects with very large heads. In this study, the head circumference was 60 cm, which represents a large head size (above the 95th percentile for men and well above average for women). We found that this was close to the upper limit of what could be accommodated by the coil. For individuals with larger head sizes, wearing wireless resonator glasses inside the coil may not be feasible. Second, the depth‐dependent SNR profile (Figure 4) indicates pronounced enhancement within approximately 5 cm of the resonators, with diminishing gains at greater depths, a characteristic of near‐field sensitivity in loop‐based receive structures [48]. To address deeper targets, such as the intracranial portion of the optic nerve, larger resonators or an increased number of resonators may be necessary. Third, the clinical value has not been extensively demonstrated. Although higher SNR typically translates to improved resolution and/or shorter scan times, further validation in larger patient cohorts is essential to establish the device's practical utility in clinical applications. Such studies would provide stronger evidence of its impact on diagnostic accuracy and patient outcomes.

## Funding

This work was supported by the National Institutes of Health (R01 EB031078, R03 EB034366, R21 EB029639, R21 EB037763, S10 OD 030389).

## Conflicts of Interest

Jason E. Moore is an employee of Philips and has no conflicts of interest.

## Supporting information


**Data S1:** mrm70326‐sup‐0001‐Figures.docx.

## Data Availability

The data that support the findings of this study are available from the corresponding author upon reasonable request.
