# Supplementary material for: Low‐Cost and Detunable Wireless Resonator Glasses for Enhanced Eye MRI With Concurrent High‐Quality Whole‐Brain MRI
Source: Magn Reson Med. 2026 Feb 24;96(1):499–507. doi: 10.1002/mrm.70326 (PMC13156439; doi:10.1002/mrm.70326)
Supplement: Supplementary file 1 — Data S1: mrm70326‐sup‐0001‐Figures.docx. [file MRM-96-499-s001.docx]

**Supplementary Materials**

The Supplementary Information includes 2 Supplementary Figures.

**Low‑Cost and Detunable Wireless Resonator Glasses for Enhanced Eye MRI with Concurrent High‑Quality Whole‑Brain MRI**

*Ming Lu ^a*^, Xiaoyue Yang ^b*^,* *Jason Moore ^a,c^, Pingping Li ^a^, Adam W. Anderson ^a,d,e^, John C. Gore ^a,d,e^, Seth A. Smith ^a,d,e^, Xinqiang Yan ^a,b,e†^*

1. Vanderbilt University Institute of Imaging Science, Vanderbilt University Medical Center, Nashville, TN, 37232, USA
2. Department of Electrical and Computer Engineering, Vanderbilt University, Nashville, TN, 37232, USA
3. Philips, Nashville, Tennessee, USA
4. Department of Biomedical Engineering, Vanderbilt University, Nashville, TN, 37232, USA
5. Department of Radiology and Radiological Sciences, Vanderbilt University Medical Center, Nashville, TN, 37232, USA

**Corresponding Author:**

Xinqiang Yan, Ph.D.

Vanderbilt University Institute of Imaging Science

1161 21^st^ Avenue South
Medical Center North, D-2205
Nashville, TN 37232-2310

Email: xinqiang.yan@vumc.org


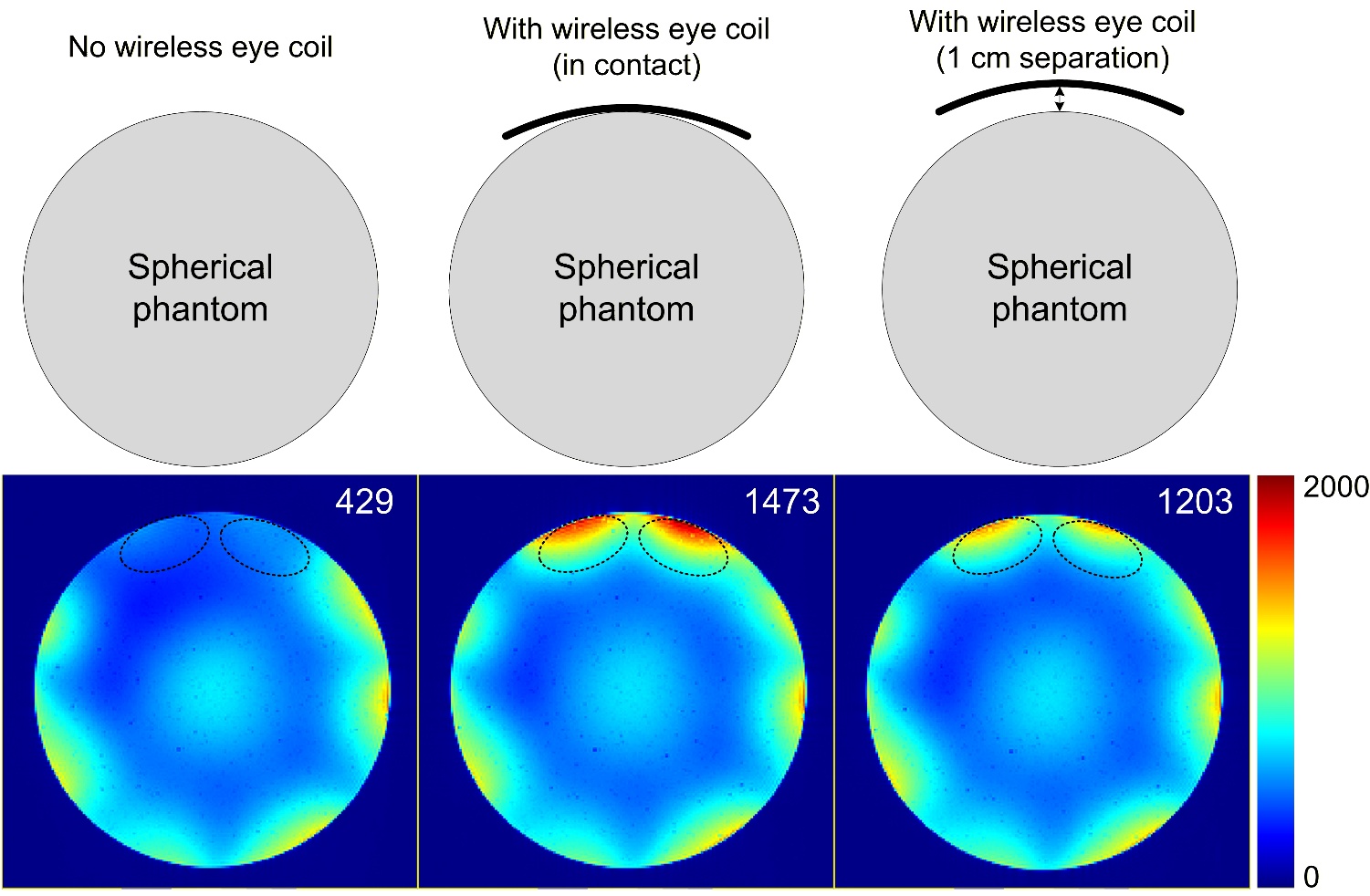


**Figure S1** Measured axial SNR maps obtained from a 17-cm-diameter spherical phantom using the Nova 2Tx/32Rx head coil under three conditions: (i) without the eye coil, (ii) with the eye coil placed as close as possible to the phantom, and (iii) with the eye coil lifted 1 cm away from the phantom. SNR maps were calculated from GRE images acquired with the following parameters: TR/TE = 1000/10 ms, FOV = 200 × 200 mm^2^, nominal flip angle = 70 degrees, voxel size = 1 × 1 mm^2^, slice thickness = 5 mm, Bandwidth = 496 Hz, number of averages = 1.


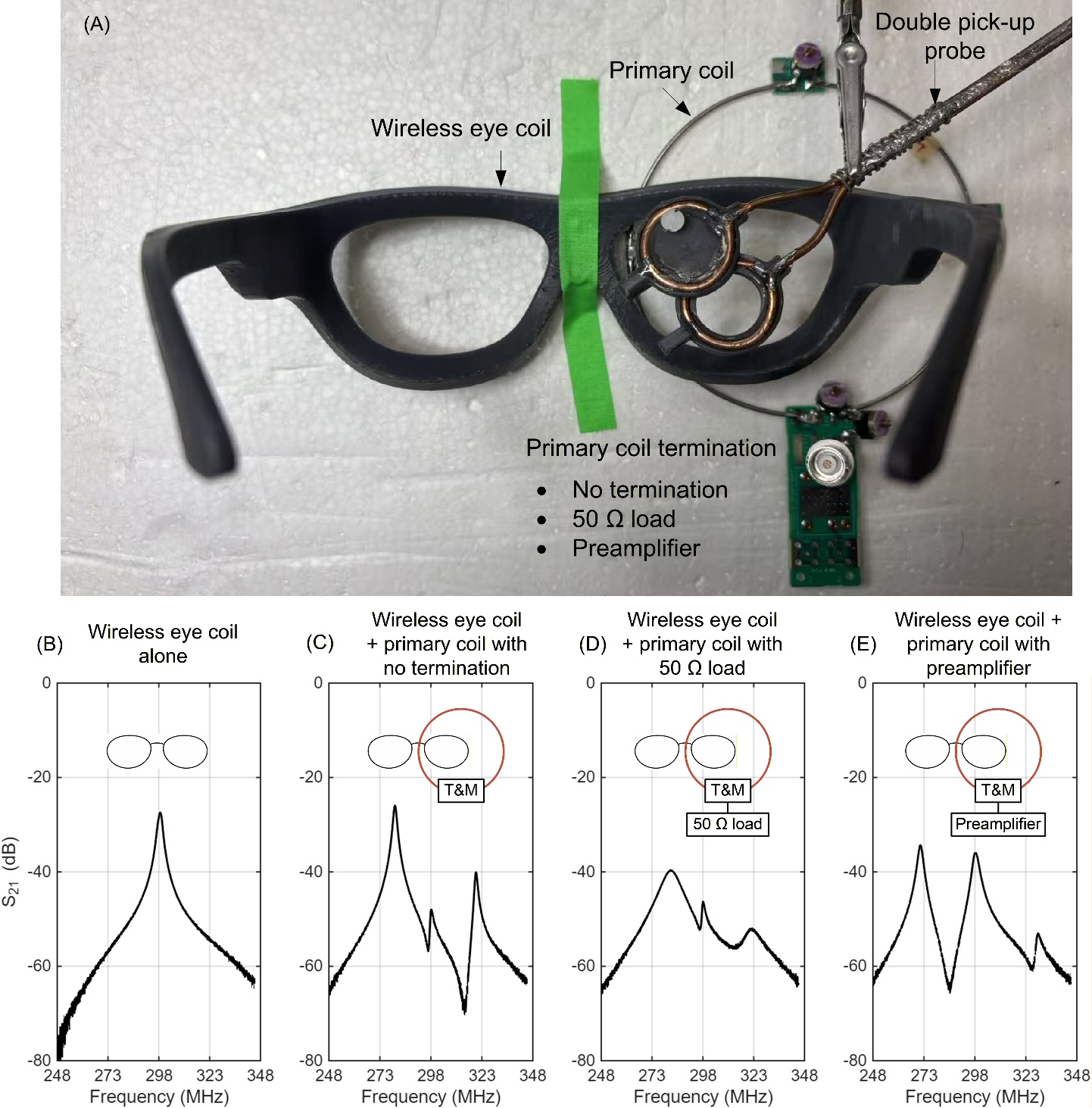


**Figure S2** Bench-measured resonance response of a single wireless resonator in the presence of a 10-cm-diameter loop coil, used to mimic the primary receive coil. Measurements were performed under three termination conditions of the 10-cm loop: (i) unterminated, (ii) terminated with 50 Ω, and (iii) terminated with a low-input-impedance preamplifier (model WMM7RP, WanTcom). (A) Measurement setup. (B–E) Bench test results under the different termination conditions.
